# Evaluation of seroprevalence and clinical and laboratory findings of patients admitted to health institutions in Gümüşhane with suspicion of Crimean-Congo hemorrhagic fever

**DOI:** 10.3906/sag-2001-82

**Published:** 2021-08-30

**Authors:** Esra GÜRBÜZ, Abdurrahman EKİCİ, Ahmet Hakan ÜNLÜ, Hasan YILMAZ

**Affiliations:** 1 Department of Infectious Diseases and Clinical Microbiology, Kelkit State Hospital, Gümüşhane Turkey; 2 Department of Parasitology, Faculty of Medicine, Van Yüzüncü Yıl University, Van Turkey; 3 Division of Veterinary, Gevaş Vocational School, Van Yüzüncü Yıl University, Van Turkey

**Keywords:** Crimean-Congo hemorrhagic fever, seroprevalence, clinical and laboratory findings

## Abstract

**Background/aim:**

To determine the seroprevalence and evaluate clinical findings and laboratory results of patients prediagnosed with Crimean-Congo hemorrhagic fever (CCHF) in Gümüşhane.

**Materials and methods:**

Included in the cross-sectional study were 362 patients (162 female, 200 male) between 0 and 94 years of age, who were followed up after receiving a preliminary diagnosis of CCHF between January 2011 and December 2019. Anamnesis, age, sex, clinical findings, laboratory results, epidemiological and clinical evaluations, severity criteria, risk factor reviews, and a comparison of the suspected negative cases with positive cases were analyzed retrospectively. Patients included in the study were evaluated as RNA-positive by polymerase chain reaction (PCR) or IgM-positive by ELISA.

**Results:**

Of the 362 patients admitted to health institutions with a preliminary diagnosis of CCHF, 242 were diagnosed as CCHF-positive (66.9%). Moreover, 196 of those CCHF-positive patients (81%) were admitted to health institutions during the summer months. Statistical analyses revealed a significant relationship between the incidence of CCHF and patients who had been in contact with animals, lived in rural areas, and had engaged in farming and animal husbandry. In addition, fever, headache, diffuse bodily pain, nausea and vomiting, diarrhea, fever of 38 °C or higher, tachycardia, elevated ALT/AST, creatine kinase (CK), and lactate dehydrogenase (LDH) levels, leukopenia, and thrombocytopenia were detected in the CCHF-positive patients. Significant relations were found between this disease and these symptoms. However, there was no significant relationship between the statistical evaluation of the disease and bloody diarrhea, bodily bruises, rash, unconsciousness, gingival bleeding, hypotension, epistaxis, petechiae, splenomegaly, ecchymosis, hematuria, maculopapular rash, gastrointestinal system complaints, anemia, or elevation of the international normalized ratio and activated partial thromboplastin time duration, separately.

**Conclusion:**

Of the 362 patients, 66.9% (242) of those who received a preliminary diagnosis of CCHF were indeed CCHF-positive in Gümüşhane. It was concluded that CCHF remains an important endemic disease in Gümüşhane. In addition, elevated ALT/AST, CK, and LDH levels, leukopenia, and thrombocytopenia in patients presenting with headache, fever, fever of 38 °C or higher, generalized body pain, nausea/vomiting, diarrhea, and tachycardia will play a pivotal role in the preliminary diagnosis of CCHF.

## 1. Introduction

Crimean-Congo hemorrhagic fever (CCHF) is a severe and fatal disease that is transmitted by ticks and often presents with bleeding symptoms in humans. The causative agent is the CCHF virus, an RNA virus of the Nairovirus lineage belonging to the family Bünyaviridae. It was first described in the Crimea in 1944, when it affected more than 200 people [1]. CCHF has been reported in more than 30 countries in Eastern Europe, Africa, the Balkans, Russia, Central Asian Republic, and the Middle East. The spread of the virus over a wide geographic area would result in a severe disease leading to high mortality in humans. The absence of a specific effective treatment and vaccine against the virus, in addition to its potential to be used as a bioterror agent or biological weapon, have made it an important human pathogen and a major public health problem worldwide [2]. In Turkey, CCHF is mainly seen in Erzurum, Erzincan, Gümüşhane, Bayburt, Tokat, Yozgat, Sivas, Amasya, Çorum, Çankırı, Bolu, Kastamonu, and Karabuk [3].

Climatic and environmental changes, increased population of ticks, movement of livestock, and transport of virus-infected ticks through migratory birds all play a role in the incidence of the disease. Transmission to humans occurs via tick bites, or contact with the tissues or blood of infected animals during the viremic period, and the blood, secretions, and mucosal or disrupted skin surfaces of infected patients with acute CCHF. Tick species of *Hyalomma marginatum*,* Amblyomma variegatum*,* Haemaphysalis punctata*,* Hyalomma anatolicum*,* Hyalomma truncatum*,* Rhipicephalus bursa* have been reported to play a role in the transmission of the virus. However, field studies conducted in Turkey since 2005 have shown that the main tick species responsible for CCHF epidemics was *Hyalomma marginatum*. The CCHF virus has the most common geographic distribution among tick-borne viruses [1,4–6].

There are 4 different stages in CCHF infection, which include incubation, and the prehemorrhagic, hemorrhagic, and convalescent periods. The incubation period of the disease varies between 3 and 14 days, depending on the route of transmission of the virus, the viral load, and the immune status of the host. After the incubation period, the prehemorrhagic stage begins with complaints like acute fever (39–41 °C), diffuse muscle pain, severe headaches, chills, trembling, nausea and vomiting, diarrhea, redness of the face and conjunctiva, photophobia, and maculopapular rash. Subsequent subcutaneous hemorrhage in the form of petechiae, purpura, and ecchymosis, and hemorrhagic symptoms, such as gum, nose, vagina, gastrointestinal tract, urinary system, lung, and brain hemorrhage, are seen in later stages of the disease. In addition, lymphocyte, monocyte, and neutrophil counts in these patients have been reported as significantly lower than in CCHF-negative patients [7]. This reduction in defense cells could have resulted from the induction of hemophagocytosis. In a previous study, the presence of effective hemophagocytosis was detected in half of the patients [8]. Liver and spleen enlargement can be seen in approximately one-third of patients. In cases with severe disease during the concession period, unconsciousness, agitation, hepatorenal insufficiency, respiratory failure, disseminated intravascular coagulopathy, shock, and coma may develop and death may occur. 

The aim of this study was to determine the seroprevalence of CCHF patients who applied to health institutions in Gümüşhane province and evaluate the relationship between CCHF and residing regions, seasons, occupations, contact with animals, some clinical findings, and laboratory results.

## 2. Materials and methods

A total of 362 patients, comprising 162 females and 200 males, between 0 and 94 years of age, who were followed up with a preliminary diagnosis of CCHF between January 2011 and December 2019 were included in this cross-sectional study. The study received approval of the local ethics committee. CCHF notification form records were analyzed from beginning to end. Anamnesis, age, sex, clinical findings, and laboratory results of the patients were obtained from the information system of the Gümüşhane Provincial Health Directorate. Epidemiological and clinical evaluations, severity criteria, risk factor reviews, and a comparison of suspected negative cases with positive cases were analyzed retrospectively.

The patients included in the study were evaluated by PCR method; RNA positivity or ELISA method; IgM positivity. In the laboratory tests, alanine aminotransferase (ALT) at 0–55 U/L, aspartate aminotransferase (AST) at 5–34 U/L, thrombocyte count at 3.5–11 10³/mm³, creatine kinase (CK) at 30–200 U/L, platelet count at 142,000%–424,000%, lactate dehydrogenase (LDH) at 0–247 mg/dL, international normalized ratio (INR) of 0.8–1.24, activated partial thromboplastin time (APTT) of 22–40 s, and anemia at 11.7–18.8 g/dL were accepted as normal.

### 2.1. Statistical analyses

In the statistical evaluation, the incidence of the disease was expressed as numbers and percentages according to the relevant categorical variables. The relationship between categorical variables was calculated using the Chi-square (χ²) test. The Z-ratio test was used to determine the average of the sample mean. Statistical significance level was taken as 5% in calculations and MINITAB (Ver:14, Minitab Inc., State College, PA, USA) statistics package program was used for calculations.

## 3. Results

Of the 362 patients included in this study, 55.3% were male (n = 200) and 44.7% were female (n = 162). The mean age was 46.2 years. The youngest of the patients admitted to health institutions and followed up with the suspicion of CCHF was just 10 days old, while the oldest was 94 years old.

In 325 of the patients (93.9%) with a history of tick bites, the tick was removed by the person themselves or their relatives, while only 21 patients opted for removal by health personnel (6.1%).

In total, 242 of the patients (66.9%) admitted to health institutions with a preliminary diagnosis of CCHF were diagnosed as CCHF-positive. Moreover, 196 of those 242 patients (81%) were admitted to the health institutions during the summer months, and 8 of the 362 patients (2.2%) admitted to health institutions with the suspicion of CCHF died as a result.

When the relationship between the incidence of CCHF and sex was examined, no significant relation was found in the statistical evaluation (p > 0.05) (Table 1).

**Table 1:** Distribution of the CCHF incidence by sex, animal contact, place of residence and occupation.

		CCHF			Total (100%)	P-value
	PositiveN, %		NegativeN, %			
Sex	Female		110 (67.9)	52 (32.1)	162	>0.05
	Male		132 (66)	68 (34)	200	
	Total		242 (66.9)	120 (33.1)	362	
Contact animals	Positive		185 (78.7)	50 (21.3)	235	<0.05
	Negative		57 (44.9)	70 (55.1)	127	
	Total		242 (66.9)	120 (33.1)	362	
Place of residence	Rural zone		206 (75.7)	66 (24.3)	272	<0.05
	Town center		36 (40)	54 (60)	90	
	Total		242 (66.9)	120 (33.1)	362	
Job	Farming/animal husbandry		149 (61.6)	47 (39.2)	196	
	Other		80 (33.1)	58 (48.3)	138	
	Student		6 (2.5)	8 (6.7)	14	
	Children (0–5 years)		6 (2.5)	7 (5.8)	13	
	Health personnel		1 (0.4)	0 (0)	1	
	Total (100%)		242	120	362	

When the relationship between the incidence of CCHF and contact with animals was investigated, a statistically significant relation was found (P < 0.05) (Table 1).

When the relationship between the incidence of CCHF and place of residence was examined, it was observed that more people living in rural areas had the disease than people living in cities. A statistically significant relationship was found between the incidence of CCHF and people living in rural areas (p < 0.05), (Table 1).

When the distribution of the incidence of CCHF according to occupations was examined, it was found that people engaged in farming and animal husbandry had a much higher rate of CCHF than those in other occupations (Table 1).

Fever, headache, generalized body pain, nausea and vomiting, diarrhea, fever of 38 °C or higher and tachycardia were observed in CCHF-positive individuals. There was a significant relation between the disease and these symptoms (p < 0.05) (Table 2). Elevated ALT/AST, CK, and LDH levels, leukopenia, and thrombocytopenia were observed in CCHF-positive individuals. There was a significant relation between the disease and these laboratory findings (p < 0.05) (Table 2). However, there was no significant relationship between the disease and bloody diarrhea, bodily bruises, rash, unconsciousness, gingival bleeding, hypotension, epistaxis, petechiae, splenomegaly, ecchymosis, hematuria, maculopapular rash, gastrointestinal system (GIS) complaints, anemia, elevation of INR, or APTT duration, separately as clinical symptoms and laboratory findings, in the statistical evaluation (p > 0.05), (Table 2, Table 3).

**Table 2:** Relationship between the incidence of CCHF and some clinical symptoms.

Clinical signs and symptoms		CCHF		Total(100%)	P-value
		PositiveN, %	NegativeN, %		
Fever	Positive	194 (72.9)	72 (27.1)	266	<0.05
	Negative	48 (50)	48 (50)	96	
Headache	Positive	197 (75.2)	65 (24.8)	262	<0.05
	Negative	45 (45)	55 (55)	100	
Diffuse body pain	Positive	217 (76.1)	68 (23.9)	285	<0.05
	Negative	25 (32.5)	52 (67.5)	77	
Nausea/vomiting	Positive	141 (73.1)	52 (26.9)	193	<0.05
	Negative	101 (59.8)	68 (40.2)	169	
Diarrhea	Positive	60 (78.9)	16 (21.1)	76	<0.05
	Negative	182 (63.6)	104 (36.4)	286	
Bloody diarrhea	Positive	7 (77.8)	2 (22.2)	9	>0.05
	Negative	235 (66.6)	118 (33.4)	353	
Bodily bruises	Positive	8 (72.7)	3 (27.3)	11	>0.05
	Negative	234 (66.7)	117 (33.3)	351	
Eruption	Positive	18 (69.2)	8 (30.8)	26	>0.05
	Negative	224 (66.7)	112 (33.3)	336	
Fever of 38 °C or above	Positive	142 (73.2)	52 (26.8)	194	<0.05
	Negative	100 (59.5)	68 (40.5)	168	
Consciousness disorder	Positive	9 (3.7)	232 (96.3)	241	>0.05
	Negative	4 (3.3)	117 (96.7)	121	
Bleeding gums	Positive	6 (2.5)	236 (97.5)	242	>0.05
	Negative	0 (0)	120 (100)	120	
Hypotension	Positive	31 (75.6)	10 (24.4)	41	>0.05
	Negative	211 (65.7)	110 (34.3)	321	
Tachycardia	Positive	27 (84.4))	5 (15.6)	32	<0.05
	Negative	215 (65.2)	115 (34.8)	330	
Epistaxis	Positive	9 (75)	3 (25)	12	>0.05
	Negative	233 (66.6)	117 (33.4)	350	
Petechiae	Positive	11 (73.3)	4 (26.6)	15	>0.05
	Negative	231 (66.6)	116 (33.4)	347	
Splenomegaly	Positive	2 (40)	3 (60)	5	>0.05
	Negative	240 (67.2)	117 (32.8)	357	
Ecchymosis	Positive	11 (84.6)	2 (15.4)	13	>0.05
	Negative	231 (66.2)	118 (33.8)	349	
Hematuria	Positive	12 (80)	3 (20)	15	>0.05
	Negative	230 (66.3)	117 (33.7)	347	
Maculopapular skin lesions	Positive	18 (78.3)	5 (21.7)	23	>0.05
	Negative	224 (66.1)	115 (33.9)	339	
Presence of GIS complaint	Positive	6 (75)	2 (25)	8	>0.05
	Negative	236 (66.7)	118 (33.3)	354	

**Table 3:** Relationship between the incidence of CCHF and some laboratory findings.

Laboratory findings		CCHF		Total(100%)	P-value
		PositiveN, %	NegativeN, %		
Anemia	Positive	47 (59.5%)	32 (40.5)	79	>0.05
	Negative	195 (68.9%)	88 (31.1)	283	
AST-ALTelevation	Positive	151 (78.6%)	41 (20.9)	192	<0.05
	Negative	91 (53.5%)	79 (46.5)	170	
Leukopenia	Positive	202 (81.5%)	46 (18.5)	248	<0.05
	Negative	40 (35.1%)	74 (64.9)	114	
CKelevation	Positive	142 (76.8)	43 (23.2)	185	<0.05
	Negative	100 (56.5)	77 (43.5)	177	
Thrombocytopenia	Positive	188 (77.7)	54 (22.3)	242	<0.05
	Negative	54 (45)	66 (55)	120	
LDHelevation	Positive	147 (77)	44 (23)	191	<0.05
	Negative	95 (55.6)	76 (44.4)	171	
INRelevation	Above 1.2	42 (65.6)	22 (34.4)	64	>0.05
	Below 1.2	200 (67.1)	98 (32.9)	298	
APTTtime	Over 15 s	74 (72.5)	28 (27.5)	102	>0.05

## 4. Discussion 

CCHF is a lethal viral causative infection that has been identified in Africa, Asia, Western Europe, and the Middle East. CCHF was the first viral hemorrhagic fever detected in Turkey thus far and the first symptomatic case was reported in 2002 in the Kelkit Valley in Tokat. Following that, reports were made regarding CCHF from Artvin, Amasya, Bayburt, Erzincan, Erzurum, Çorum, Çankırı, Kastamonu, Sivas, and Yozgat. In recent years, the disease areas have widened and cases have been reported from every region of Turkey. A significant increase in CCHF cases has been observed in Turkey since 2002. Most cases of CCHF were observed in the Tokat, Yozgat, Çorum, Sivas, Kastamonu, Karabuk, Gümüşhane, Erzurum, Amasya, Çankırı, Giresun, and Samsun provinces of Turkey [9].

The fact that deaths were caused by tick bites in the written and visual literature has resulted in serious concern, sensitivity, and awareness against ticks in society. In parallel, the number of patients admitted to the emergency department has increased over the years. When the number of CCHF cases was examined, it is observed that there were 150 in 2002–2003, 249 in 2004, 266 in 2005, 438 in 2006, 717 in 2007, and 1315 in 2008 [9]. According to data from the Ministry of Health, a total of 9787 cases of CCHF were reported in Turkey between 2002 and 2015 [10].

In the present study, of the 362 patients who were followed up after receiving a preliminary diagnosis of CCHF, 242 were diagnosed as CCHF-positive (66.9%). The fact that CCHF was first reported in the Black Sea region of Turkey, and that the positivity rate was high in this study, led to the conclusion that CCHF is still an important endemic disease in Gümüşhane [9].

Although the fight against ticks is very important in the protection against CCHF, it is very difficult to achieve. Ticks can suck blood by attacking humans and animals in all biological stages except in the egg stage. Feeding is essential in order to continue their development and maintain generation. The basis of protection from ticks is to avoid known tick habitats and the application of pesticides to these areas [11]. 

Agricultural and livestock workers (animal caretakers, milkers, and shearing workers, shepherds, butchers, slaughterhouse workers), veterinarians, those in contact with sick animals, and health personnel working in endemic areas (due to the possibility of contact with acute patients), soldiers, campers/picnickers, and leather factory workers are at high risk. According to the data of the Ministry of Health, 67% of infected patients were engaged in agriculture and/or animal husbandry [4]. In a study conducted using data obtained from patients diagnosed with CCHF between 2006 and 2012 in Bolu, Turkey, 48.6% of the patients were housewives, 27% were livestock workers, 10.8% were farmers, 5.4% were health workers, and 8.1% were in other occupational groups [12]. In a study conducted in eastern Turkey, it was determined that 98.4% of CCHF-positive patients resided in rural areas and 72.1% had a history of contact with ticks [13]. Likewise, in a study conducted in Georgia, it was found that the disease was mostly found in farmers and people who lived in rural areas [14]. In a study investigating the molecular and clinical epidemiology of CCHF in Oman [15], the main risks of infection for contracting CCHF were animal contact (73%) and butchery (88%). When the distribution of ticks according to occupations was examined in the current study, it was found that 54.1% of the 362 patients were engaged in farming and animal husbandry, 38.1% were from other occupational groups, 3.9% were students, 3.6% were children, and 0.3% were health personnel. CCHF positivity was highest among 61.6% of those engaged in farming and animal husbandry, while it was 33% in other occupational groups, 2.5% in students and children, and 0.4% in health personnel. In addition, a statistically significant relationship was found between the incidence of CCHF and patients with a history of contact with animals and people living in rural areas (Table 1). Therefore, it was determined that it is very important to raise awareness about CCHF, especially for people living in endemic and rural areas, involved in livestock and farming, and in contact with animals. 

In the present study, 93.9% of the 346 patients with a history of tick attachment removed the tick themselves or it was removed by their relatives, while the rate of removal by health personnel was only 6.1%. If ticks are not properly removed, they cause tissue damage. There is a risk that the capitulum of the tick will rupture and remain in the skin. In addition, incorrect removal of the tick could also increase the probability of transmission of the disease, even if the host has not been infected yet [16]. In cases of tick bites in endemic areas, individuals should seek medical assistance at a health facility without delay and awareness should be raised about the removal of ticks in accordance with correct methods.

To summarize some of the studies carried out in line with the data obtained from clinical findings of CCHF, in a study investigating the relationship between headache and severity of CCHF, it was found that headache was more severe in positive patients than in the control group and had similar characteristics to the pain experienced by migraine patients [17]. In another study conducted in Kastamonu, it was found that headache and nausea/ vomiting associated with leukopenia should be taken into consideration in patients presenting with suspicion of CCHF [18]. In a study conducted in Amasya, headache, fever, general body pain, nausea and vomiting, and fatigue were among the clinical symptoms that should be considered in CCHF [19]. In another study, high fever was shown to be an important symptom in CCHF patients [14]. In the current study, 75.2% of patients with headache, 72.9% of patients with fever, 73.2% of patients with fever of 38 °C or higher, 76.1% of patients with diffuse body pain, 73.1% of patients with nausea and vomiting, 78.9% of patients with diarrhea, and 84.4% of patients with tachycardia were diagnosed with CCHF. A statistically significant relation was found between CCHF and clinical symptoms of headache, fever, fever of 38 °C or higher, diffuse body pain, nausea and vomiting, diarrhea, and tachycardia (p < 0.05) (Table 2). It was concluded that CCHF should be considered in individuals living or traveling to endemic areas who present at health institutions with the indicated clinical symptoms.

A Pakistani case report on CCHF showed that 4 of 6 patients had GIS complaints without any signs of bleeding before the disease was suspected [20]. In another study, a statistically significant relationship was found between CCHF and epistaxis [21]. In the current study, no significant relationship was found between CCHF and epistaxis, GIS, bloody diarrhea, bodily bruising, rash, unconsciousness, gingival bleeding, hypotension, petechiae, splenomegaly, ecchymosis, hematuria, maculopapular rash, anemia, elevation of INR, or duration of APTT (p > 0.05). There are 4 different stages of CCHF infection [7]. Most of the patients admitted in this study with suspicion of CCHF were thought to be in the first 2 stages of incubation and prehemorrhagic period. Epistaxis and GIS complaints occur later in the disease. Therefore, it was thought that there was no significant relationship between CCHF and these complaints.

To summarize some of the studies carried out in line with the data obtained from laboratory findings in CCHF, it was reported by Büyüktuna et al. that routine monitoring of AST levels may help patients with bleeding conditions and additionally reduce liver damage [22]. In addition, it was concluded that ferritin levels could be a potential biomarker that would be useful in monitoring patients. As a result of a study conducted using data obtained from patients diagnosed with CCHF in a hospital in Kastamonu, 41% of 76 CCHF-suspected patients were diagnosed as CCHF-positive [9]. It was concluded that CCHF should be considered in cases of elevated thrombocytopenia, ALT/AST, and LDH levels. In a study conducted in Amasya, Turkey, 281 patients admitted to a hospital were diagnosed with CCHF and it was concluded that leukopenia, thrombocytopenia, and elevated ALT/AST, CK and LDH levels should be taken into consideration in the diagnosis of the disease [19]. In a study of 240 patients admitted to a hospital in Çorum, Turkey, with suspected CCHF, patients were diagnosed as CCHF-positive via the RT-PCR method with regards to the laboratory findings, leukopenia, thrombocytopenia, and AST and potassium levels, which were statistically significant between the patients and positive diagnoses [21]. In a study conducted in Georgia, the laboratory findings of CCHF-positive patients revealed that the most prominent symptoms were intoxication and hemorrhagic symptoms, thrombocytopenia, and high creatine and aminotransferase levels [14]. In the current study, 78.6% of patients with elevated ALT/AST levels, 81.5% of patients with leukopenia, 76.8% of patients with elevated CK levels, 77.7% of patients with thrombocytopenia, 77.7% of patients with thrombocytopenia, and 77% of patients with elevated LDH levels were diagnosed as CCHF-positive. A statistically significant relation was found between CCHF and the laboratory findings; elevated ALT/AST, CK, and LDH levels, leukopenia, and thrombocytopenia (p < 0.001) (Table 3). No statistically significant relationship existed between CCHF and anemia, elevated INR or duration of APTT (p > 0.05). Therefore, CCHF should be kept in mind when diagnosing people living in or visiting endemic areas, especially in patients with elevated ALT/AST, CK, and LDH levels, leukopenia, and thrombocytopenia.

In a study investigating the relationship between CCHF and climate characteristics, it was found that there was a significant relationship between moisture and precipitation data and the number of patients admitted to hospitals with CCHF complaints [23]. Therefore, it is important to consider climatic characteristics in regions where CCHF has been diagnosed. In the current study, 81% of patients who were CCHF-positive presented at health institutions during the summer months. This is significant because CCHF is a disease transmitted by ticks, which become specifically more active as the weather warms up; thus; the risk of contact with people increases. In addition, people are also more likely to frequent places where ticks are active during the summer months, enhancing their risk of infection.

As a result, CCHF has been reported from almost every region of Turkey, with varying frequency. In the summer months, when CCHF incidence increases, the history of animal contact and tick bites should be questioned and examination specifically for ticks/tick bites should be performed. The public should be made aware of the importance of removal of ticks by expert health personnel. Raising awareness about CCHF should be performed via visual and media announcements, and especially to those working in farming and animal husbandry, and living in rural areas where CCHF is endemic. Patients admitted to health institutions with complaints including headache, fever, fever of 38 °C or higher, diffuse bodily pain, nausea and vomiting, diarrhea, or tachycardia, with laboratory findings such as elevated ALT/AST, CK, and LDH levels, leukopenia, and thrombocytopenia, should be carefully examined for CCHF, as are the key markers in its preliminary diagnosis. In addition, it will be of great importance to take timely precautions for protection against ticks, especially during the summer months when ticks are more active.

## Informed consent

Ethics approval was obtained from the Van Yüzüncü Yıl University Non-invasive Ethics Committee (2019/14-02).
